# Supplementary material for: Protocols Targeting Afferent Pathways via Neuromuscular Electrical Stimulation for the Plantar Flexors: A Systematic Review
Source: Sensors (Basel). 2023 Feb 20;23(4):2347. doi: 10.3390/s23042347 (PMC9967278; doi:10.3390/s23042347)
Supplement: Supplementary file 1 [file sensors-23-02347-s001.zip › sensors-2101968-supplementary.pdf]

Supplementary Materials

Appendix S1: Search strategies and number of retrieved papers for each database.

Database	Retrieved Papers	Keywords
Cochrane Library	58	(“neuromuscular electrical stimulation”): ti,ab,kw AND (“tibial nerve*” OR “triceps surae*” OR “soleus*” OR “gastrocnemious*” OR “calf*” OR “shank*” OR “plantar flex”):ti,ab,kw in Trials
PubMed	76	“neuromuscular electrical stimulation*”[All Fields] AND (“tibial nerve*”[All Fields] OR “triceps surae*”[All Fields] OR “soleus*”[All Fields] OR “gastrocnemious*”[All Fields] OR “calf*”[All Fields] OR “shank*”[All Fields] OR “plantar flex*”[All Fields]) Filters: Humans, English
Scopus	111	(TITLE-ABS-KEY(“neuromuscular electrical stimulation*”)) AND (TITLE-ABS-KEY(“tibial nerve*” OR “triceps surae*” OR “soleus*” OR “gastrocnemious*” OR “calf*” OR “shank*” OR “plantar flex*”)) AND (LIMIT-TO (PUBSTAGE,”final”)) AND (LIMIT-TO (DOCTYPE,”ar”)) AND (LIMIT-TO (EXACTKEYWORD,”Human”) OR LIMIT-TO (EXACTKEYWORD,”Humans”)) AND (LIMIT-TO (LANGUAGE,”English”)) AND (LIMIT-TO (SRCTYPE,”j”))
Web of Science	392	(ALL= (neuromas``cular electrical stimulation*)) AND (ALL=(“tibial nerve*” OR “triceps surae*” OR “soleus*” OR “gastrocnemious*” OR “calf*” OR “shank*” OR “plantar flex*”)) *AND* LANGUAGE: (English) *AND* DOCUMENT TYPES: (Article)

Appendix S2. Quality assessment of the studies in chronological order (date of publication) according to Effective Public Health Practice Project (EPHH).

							Intervention integrity			Analyses				
Study	A	B	C	D	E	F	Q1	Q2	Q3	Q1	Q2	Q3	Q4	Global
Collins, 2002 [5]	2	2	3	2	1	1	<60%	Yes	No	Org./Inst.	Org./Inst.	Yes	No	2
Baldwin, 2006 [18]	2	2	1	2	1	1	80-100%	No	No	Org./Inst.	Org./Inst.	Yes	No	1
Klakowicz, 2006 [20]	2	2	2	2	1	2	80-100%	Yes	No	Org./Inst.	Org./Inst.	Yes	No	1
Dean, 2007 [8]	2	2	1	2	1	2	80-100%	Yes	No	Org./Inst.	Org./Inst.	Yes	No	1
Dean, 2008 [37]	2	2	1	2	1	2	80-100%	Yes	No	Org./Inst.	Org./Inst.	Yes	No	1
Lagerquist, 2009 [41]	3	2	2	2	1	1	80-100%	Yes	No	Org./Inst.	Org./Inst.	Yes	No	2
Lagerquist, 2010 [11]	2	2	1	2	1	2	80-100%	Yes	No	Org./Inst.	Org./Inst.	Yes	No	1
Bergquist, 2011 [17]	2	2	1	2	1	2	80-100%	Yes	No	Org./Inst.	Org./Inst.	Yes	No	1
Clair, 2011 [35]	2	2	2	2	1	2	80-100%	Yes	No	Org./Inst.	Org./Inst.	Yes	No	1
Lagerquist, 2012 [12]	2	2	1	2	1	1	80-100%	Yes	No	Org./Inst.	Org./Inst.	Yes	No	1
Dean, 2014 [38]	2	2	1	2	1	2	80-100%	Yes	No	Org./Inst.	Org./Inst.	Yes	No	1
Doix, 2014 [21]	2	2	1	2	1	1	80-100%	Yes	No	Org./Inst.	Org./Inst.	Yes	No	1
Neyroud, 2014 [42]	2	2	1	2	1	1	80-100%	Yes	No	Org./Inst.	Org./Inst.	Yes	No	1
Papaiordanidou, 2014 [44]	2	2	1	2	1	1	80-100%	Yes	No	Org./Inst.	Org./Inst.	Yes	No	1
Papaiordanidou, 2014 [43]	2	2	1	2	1	1	80-100%	Yes	No	Org./Inst.	Org./Inst.	Yes	No	1
Regina Dias Da Silva, 2015 [45]	2	2	1	2	1	1	80-100%	Yes	No	Org./Inst.	Org./Inst.	Yes	No	1
Wegrzyk, 2015 [34]	2	2	1	2	1	1	80-100%	Yes	No	Org./Inst.	Org./Inst.	Yes	No	1
Wegrzyk, 2015 [47]	2	2	1	2	1	1	80-100%	Yes	No	Org./Inst.	Org./Inst.	Yes	No	1
Martin, 2016 [26]	2	2	1	2	1	2	80-100%	Yes	No	Org./Inst.	Org./Inst.	Yes	No	1
Wegrzyk, 2017 [15]	2	2	1	2	1	2	80-100%	Yes	No	Org./Inst.	Org./Inst.	Yes	No	1
Cheng, 2017 [36]	2	2	1	2	1	1	80-100%	Yes	No	Org./Inst.	Org./Inst.	Yes	No	1
Grosprêtre, 2017 [39]	2	2	1	2	1	1	80-100%	Yes	No	Org./Inst.	Org./Inst.	Yes	No	1
Grosprêtre, 2018 [40]	2	2	1	2	1	1	80-100%	Yes	No	Org./Inst.	Org./Inst.	Yes	No	1
Mani, 2018 [32]	2	1	1	2	1	1	80-100%	Yes	No	Org./Inst.	Org./Inst.	Yes	No	1
Neyroud, 2018 [7]	2	2	1	2	1	1	80-100%	Yes	No	Org./Inst.	Org./Inst.	Yes	No	1
Neyroud 2019 [16]	2	1	1	2	1	1	80-100%	Yes	No	Org./Inst.	Org./Inst.	Yes	No	1
Vitry, 2019 [13]	2	2	1	2	1	1	80-100%	Yes	No	Org./Inst.	Org./Inst.	Yes	No	1
Vitry, 2019 [46]	2	2	1	2	1	1	80-100%	Yes	No	Org./Inst.	Org./Inst.	Yes	No	1
Vitry, 2019 [30]	2	1	1	2	1	1	80-100%	Yes	No	Org./Inst.	Org./Inst.	Yes	No	1
Bouguetoch, 2021 [29]	2	1	1	2	1	1	80-100%	Yes	No	Org./Inst.	Org./Inst.	Yes	No	1
Donnelly, 2021 [31]	2	1	2	2	1	1	80-100%	Yes	No	Org./Inst.	Org./Inst.	Yes	No	1
Espeit, 2021 [33]	2	1	1	2	1	1	80-100%	Yes	No	Org./Inst.	Org./Inst.	Yes	No	1

Abbreviations: A: Selection bias; B: Study design; C: Confounders; D: Blinding; E: Data collection methods; F: Withdrawals and dropouts; 1: Strong; 2: Moderate; 3: Weak; Org./Inst: Organization/Institution.
